# Synthesis and Characterization of Tissue Plasminogen Activator—Functionalized Superparamagnetic Iron Oxide Nanoparticles for Targeted Fibrin Clot Dissolution

**DOI:** 10.3390/ijms18091837

**Published:** 2017-08-24

**Authors:** Susanne Heid, Harald Unterweger, Rainer Tietze, Ralf P. Friedrich, Bianca Weigel, Iwona Cicha, Dietmar Eberbeck, Aldo R. Boccaccini, Christoph Alexiou, Stefan Lyer

**Affiliations:** 1 Department of Otorhinolaryngology—Head and Neck Surgery, Section of Experimental Oncology and Nanomedicine (SEON), Else Kröner-Fresenius-Stiftungsprofessur, Universitätsklinikum Erlangen, Friedrich-Alexander-Universität Erlangen-Nürnberg, 91054 Erlangen, Germany; susi_heid1@yahoo.de (S.H.); harald.unterweger@uk-erlangen.de (H.U.); rainer.tietze@uk-erlangen.de (R.T.); Ralf.friedrich@uk-erlangen.de (R.P.F.); bianca.weigel@uk-erlangen.de (B.W.); Iwona.cicha@uk-erlangen.de (I.C.); stefan.lyer@uk-erlangen.de (S.L.); 2 Physikalisch-Technische Bundesanstalt, Braunschweig und Berlin, 10587 Berlin, Germany; Dietmar.eberbeck@ptb.de; 3 Institute of Biomaterials, Department of Materials Science and Engineering, University Erlangen-Nuremberg, 91058 Erlangen, Germany; aldo.boccaccini@fau.de

**Keywords:** protein binding, activated ester reaction, fibrinolysis, tissue plasminogen activator, superparamagnetic iron oxide nanoparticles, drug targeting

## Abstract

Superparamagnetic iron oxide nanoparticles (SPIONs) have attracted great attention in many biomedical fields and are used in preclinical/experimental drug delivery, hyperthermia and medical imaging. In this study, biocompatible magnetite drug carriers, stabilized by a dextran shell, were developed to carry tissue plasminogen activator (tPA) for targeted thrombolysis under an external magnetic field. Different concentrations of active tPA were immobilized on carboxylated nanoparticles through carbodiimide-mediated amide bond formation. Evidence for successful functionalization of SPIONs with carboxyl groups was shown by Fourier transform infrared spectroscopy. Surface properties after tPA immobilization were altered as demonstrated by dynamic light scattering and ζ potential measurements. The enzyme activity of SPION-bound tPA was determined by digestion of fibrin-containing agarose gels and corresponded to about 74% of free tPA activity. Particles were stored for three weeks before a slight decrease in activity was observed. tPA-loaded SPIONs were navigated into thrombus-mimicking gels by external magnets, proving effective drug targeting without losing the protein. Furthermore, all synthesized types of nanoparticles were well tolerated in cell culture experiments with human umbilical vein endothelial cells, indicating their potential utility for future therapeutic applications in thromboembolic diseases.

## 1. Introduction

The clinical manifestations of acute atherothrombotic events, including myocardial infarction or ischemic stroke, constitute the most common cause of death worldwide and are the leading cause of disability in adults. Thrombosis, the formation of life-threatening clots that obstruct blood vessels supplying the vital organs, is a key pathological feature in many cardiovascular disorders, and its prevalence continues to rise [1,2]. From the viewpoint of intensive care medication, rapid recanalization of an occluded artery is essential to reduce mortality and improve outcomes. In patients with acute ischemic stroke, intravenous thrombolysis remains the treatment of choice, whereby recombinant tissue plasminogen activator (tPA) is the only drug approved for acute ischemic stroke since 1996 [3]. Although no other medication has demonstrated comparable efficacy, the use of tPA is very limited by both the narrow eligibility and administration window, as well as by the risk of thrombolysis-related intracerebral hemorrhagic complications [4], which occur in about 6% of patients, resulting in nearly 50% mortality [5,6]. Additionally, in some patients eligible for tPA treatment, the outcome is poor when occlusion occurs in large arteries (internal carotid artery or middle cerebral artery), even if tPA is administered via the intraarterial route [7]. Hence, there is an urgent need for an improved therapeutic regimen and targeted application of this very potent fibrinolytic drug.

**Figure 1:**
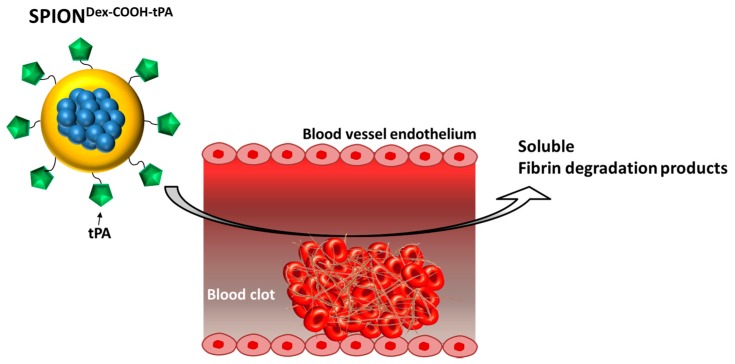
Graphical scheme of the research idea. Tissue plasminogen activator (tPA)-loaded iron oxide nanoparticles (SPION^Dex−COOH−tPA^) can target blood clots via magnetic guidance and induce local fibrinolysis.

For this purpose, thrombus targeting nanosystems could serve as carriers for enhanced delivery of tPA to the thrombi in order to increase its effective local concentrations. A nanoparticle-based drug delivery approach has been shown to be effective for the targeted accumulation of active agents in the diseased region [8,9,10,11]. Most studies have focused on anti-tumor drugs, and multiple nanoscale delivery systems are currently being developed that address cancer therapy. Many of those particle formulations have been decorated with specific recognition molecules enabling tissue-specific accumulation and drug release. However, it is very challenging to develop nanosystems that are invisible to the reticuloendothelial system with affinity to the target area. Apart from molecular recognition, magnetic attraction of iron oxide nanoparticles is considered a promising method for drug delivery. In particular, superparamagnetic iron oxide nanoparticles (SPIONs) are considered as suitable tools for this approach. SPIONs consist of an iron oxide core, often coated with organic materials such as fatty acids, polysaccharides or polymers to improve colloidal stability and to prevent separation into particles and carrier medium [12]. Accumulation of magnetic nanoparticles was previously demonstrated in a multitude of experimental magnetic field applications. Commonly, a permanent magnet is placed in the targeted area, for example, ferric steel implants, which were placed in the subarachnoid space of an in vitro human spine model [13]. An interesting concept in the context of cardiovascular diseases includes diluted microferrimagnetic wires implanted within blood vessels, which, under an externally applied magnetic field, provide specific enrichment of simultaneously administered ferromagnetic nanoparticles [14]. An example of successful application of magnetically targeted drug delivery to a tumor was reported by Tietze et al. in 2013 [8], whereby intra-arterially administered drug-loaded SPIONs accumulated in tumor tissue by externally applied magnetic fields generated by a strong electromagnet. Magnetic targeting in this model led to 56-fold enrichment of the delivered drug dose in comparison to conventional systemic application, which was related to significant improvement of therapeutic outcomes. It must be noted, however, that the majority of the previous studies utilizing magnetic drug targeting approach addressed drug accumulation under microcirculatory conditions. In the present study, we aimed to combine the fibrinolytic effects of tPA with the active targeting abilities of SPIONs (Figure 1). For this purpose, we developed a new nanoscale drug delivery system, based on biocompatible dextran-coated SPIONs carrying sufficient payloads of tPA, for local thrombolysis by means of magnetic drug targeting.

## 2. Results

### 2.1. Physicochemical Properties

**Figure 2:**
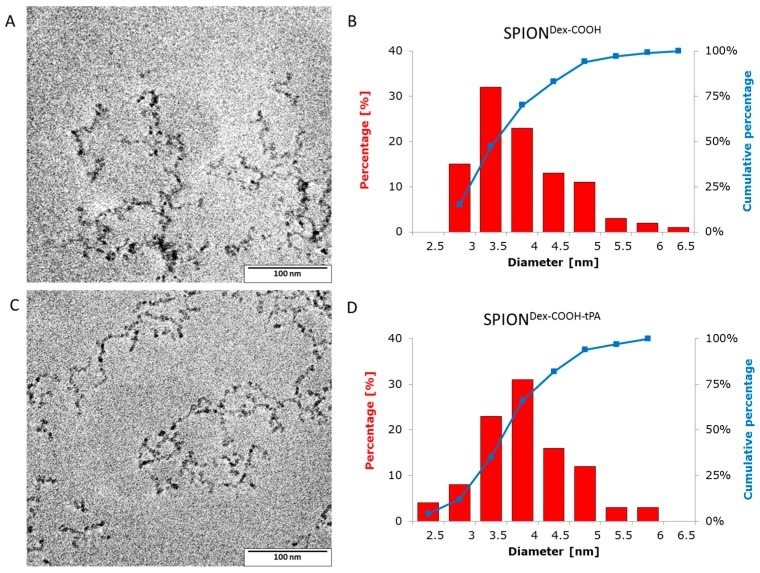
(**A**) Transmission electron microscopy (TEM) overview image of SPION^Dex−COOH^ with covalently grafted tPA (SPION^Dex−COOH^) and (**B**) corresponding size distribution of magnetite core particles, which was obtained by measuring 100 core particles with the ImageJ software. (**C**,**D**) depict corresponding results for SPION^Dex−COOH^ with covalently grafted tPA (SPION^Dex−COOH−tPA^). The narrow size distributions for both particles show no significant changes after binding of tPA.

Dextran-coated SPIONs (SPION^Dex^) were synthesized in a cold gelation process and functionalized with carboxyl groups (SPION^Dex−COOH^) via monochloroacetic acid as described previously [15]. Subsequently, tPA was bound to the particles via adsorption or covalent binding due to a 1-Ethyl-3-(3-dimethylaminopropyl) carbodiimide (EDC)/*N*-Hydroxysuccinimide (NHS) ester. The particle core diameters after functionalization and tPA immobilization, measured by transmission electron microscopy (TEM), are shown in Figure 2. Additionally, the crystallite size distributions were examined by measuring the diameter of 100 randomly selected particles using ImageJ software. In the representative images in Figure 2A,C, particles show a nearly spherical morphology. The size-distribution histograms (Figure 2B,D) show iron oxide core diameters in the range between 2.5 and 6.5 nm, with mean core diameters of 3.7 ± 0.8 nm for SPION^Dex–COOH^ and 3.8 ± 0.8 nm for SPION^Dex−COOH−tPA^. For both samples, these crystallite size distributions were quite narrow and had the same order of magnitude, independent of carboxymethylation or covalent tPA bonding.

Using dynamic light scattering (DLS), we observed a mean hydrodynamic particle cluster size of 65 ± 3 nm for SPION^Dex^. After functionalization with carboxyl groups, it increased to 173 ± 9 nm (see Table 1). Since the tPA linking reaction was performed in 2-(*N*-morpholino)ethanesulfonic acid (MES) buffer and the ionic strength affects the size of the solvent layer covering the particles, unloaded SPION^Dex−COOH^ in the same environment served as a control. Linking of protein to SPION^Dex−COOH^ (166 ± 4 nm in buffer) with carbodiimide coupling slightly increased the hydrodynamic size to 170 ± 6 nm, which may be attributed to the tPA binding, but it is not real proof of successful binding. Using different amounts of tPA, no significant change in the particle diameter was observed.

**Figure 3:**
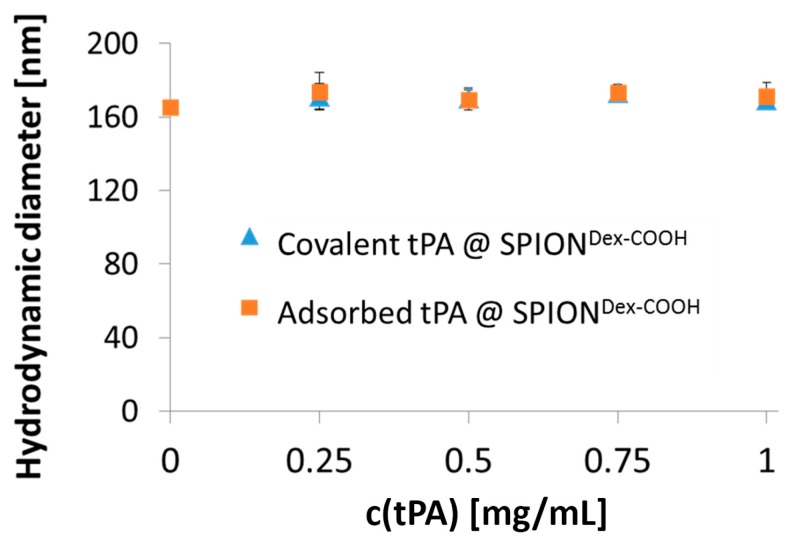
Hydrodynamic size of carboxymethylated SPIONs dependent on covalently bound or adsorbed tPA with different concentrations. Shown are the mean values of *n* = 3 with standard deviations.

**Figure 4:**
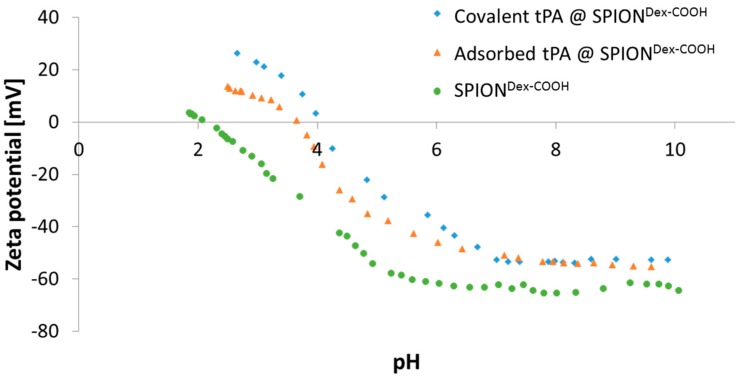
Change of ζ potential due to binding of tPA to SPION^Dex−COOH^ at a tPA concentration of 0.5 mg/mL. Shown are the mean values of *n* = 3 without standard deviations for better visibility.

As shown in Figure 3, nanoparticle size was independent of the chosen binding mechanism (covalent vs. adsorptive). The synthesized particles were characterized by a high stability in aqueous medium even after tPA binding, and no sedimentation or further agglomeration was detectable after 5 weeks of storage [16]. Carboxymethylation of SPION^Dex^ led to a modification of the ζ potential, which changed the particle’s surface properties and enabled electrosteric stability. As presented in Figure 4, an aqueous dispersion of the synthesized colloid possessed a strongly negative ζ potential at physiological pH. Regarding the pH dependency, the carboxyl groups led to a negative ζ potential over the whole pH range between pH 2 and 10. With decreasing pH, the effective surface charge also declined, but no sedimentation or precipitation was observed. Independent of the pH change, the nanoparticle diameter measured with DLS stayed almost constant [16].

**Figure 5:**
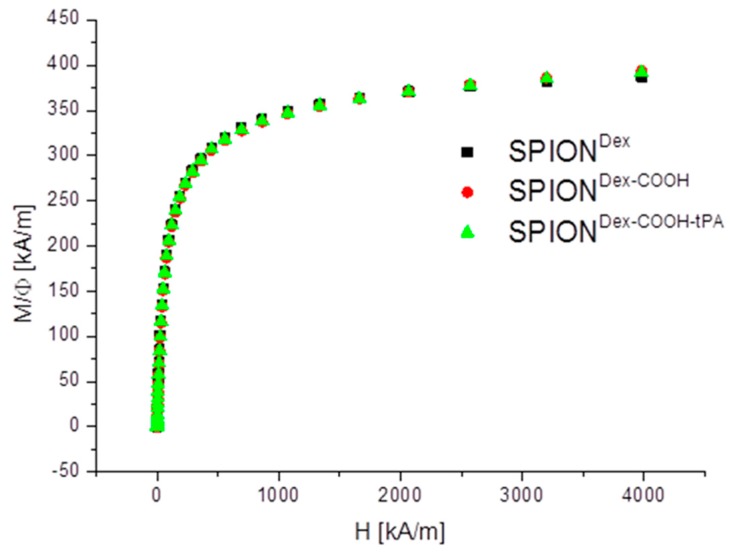
Magnetization curves of SPION^Dex^, SPION^Dex−COOH^ and SPION^Dex−COOH−tPA^. Neither the functionalization nor tPA binding led to an alteration of the M(H) data.

Magnetic properties are an important feature for the delivery of SPION-bound tPA to the site of thrombosis with the aid of an external magnetic field. Therefore, magnetization curves of SPION^Dex^, SPION^Dex−COOH^ and SPION^Dex−COOH−tPA^ were determined using Superconducting quantum interference device (SQUID) measurements, as depicted in Figure 5. All samples showed superparamagnetic behavior, with no hysteresis and no remanence, and were characterized by a saturation magnetization of about 391 ± 3.5 kA/m.

**Figure 6:**
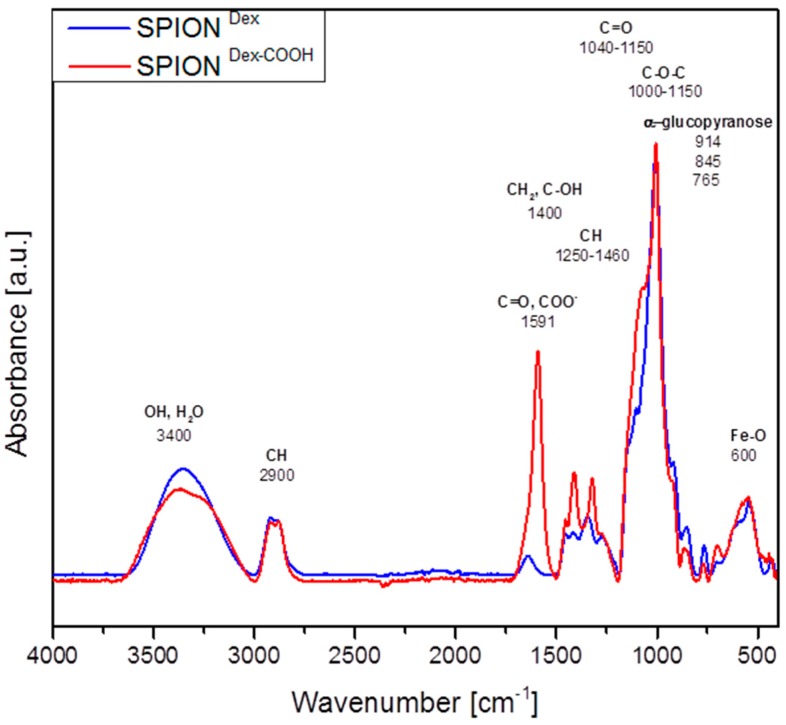
Fourier transform infrared spectroscopy (FTIR) spectra of SPION^Dex^ and SPION^Dex−CooH^.

An established method for analyzing the chemical structure of the coated iron oxide nanoparticles is Fourier transform infrared spectroscopy (FTIR). Here, the measurements were evaluated for –COOH functionalized nanoparticles in the range from 4000 cm^−1^ to 400 cm^−1^ (Figure 6). All nanoparticles featured a strong peak at around 550 cm^−1^, related to Fe–O surface vibrations of the iron oxide core [17]. The α-glucopyranose ring deformation modes at 765, 845 and 914 cm^−1^ confirmed the presence of dextran. C–O–C and C=O vibrational modes can be detected in the range between 1000–1150 cm^−1^ and 1040–1150 cm^−1^, respectively [18]. Peaks at 1400 cm^−1^ appear due to CH_2_ and C–O–H deformations [19]. Furthermore, absorption bands of C–H vibrations in dextran arise between 1250–1460 cm^−1^ as well as around 2900 cm^−1^ [20]. O–H deformation and stretching modes of hydroxyl groups are located at 1600 and 3400 cm^−1^. They exist in the dextran coating, but are often overlapped by physically adsorbed water [20].

Subsequent carboxyl functionalization of dextran-coated SPIONs was clearly demonstrated with the new occurring antisymmetric (COO)^−^ and C=O stretch modes at 1591 cm^−1^ [18]. In addition, the absorption band around 3400 cm^−1^ was lowered, as -OH groups of dextran were replaced by–COOH groups. Due to the low molar ratio of tPA in relation to the polymer-coated particles, FTIR measurements did not detect the presence of tPA on the particles. As a result, it was necessary to use other methods to analyze the efficacy of tPA binding or adsorption to the nanoparticles (see Section 2.2).

### 2.2. Bonding of tPA to Carboxymethylated Particles

**Figure 7:**
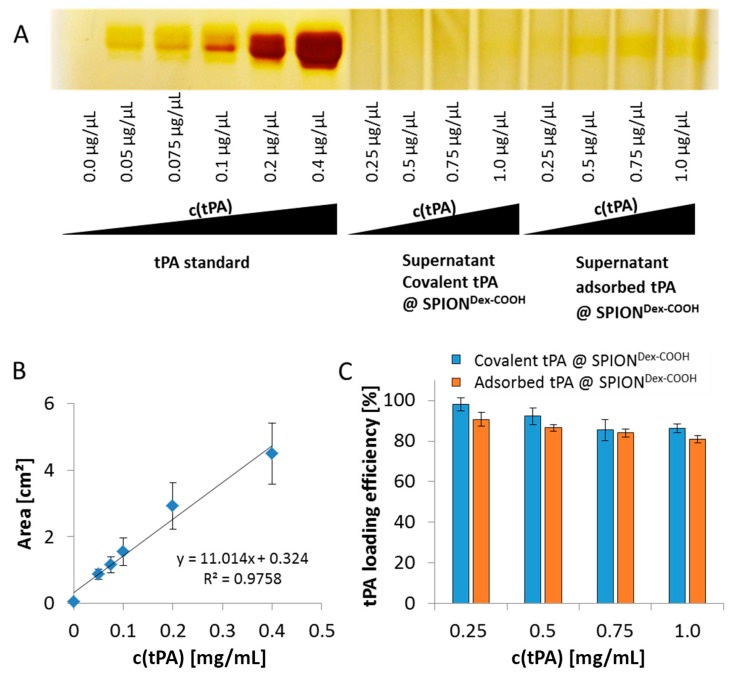
(**A**) Silver staining after sodium dodecyl sulfate polyacrylamide gel electrophoresis (SDS-PAGE) of the supernatants of covalently bound and adsorbed diafiltrated tPA on SPION^Dex−COOH^. The remaining tPA amounts in the supernatants of the reactions were analyzed with ImageJ to indirectly calculate the binding efficiencies; (**B**) The area of pure tPA’s bands were utilized for calculation of the respective binding efficiencies; (**C**) Binding efficiencies of covalently and non-covalently linked tPA to carboxymethylated iron oxide nanoparticles dependent on the tPA concentration. Shown are the mean values of *n* = 3 with standard deviations.

Electrophoresis was used to evaluate the tPA binding efficiency (Figure 7A). It was performed using the supernatants of the respective reaction mixtures, and the lack of protein bands in these samples was an indication of the successful immobilization of tPA on particles. The signal of the standard curve correlated linearly with the used tPA concentration (Figure 7B) and was used to determine the tPA loading efficiency (Figure 7C). At a tPA concentration of 0.25 mg/mL, 98 ± 3% of the protein was found to be covalently bound to SPIONs. Increasing the amount of protein led to a decrease in the binding efficiency to 86 ± 2%. A similar effect was observed in case of adsorbed tPA, with binding efficacy values decreasing from 91 ± 4% to 81 ± 2%.

An additional effect of protein attachment was observed in pH-dependent electrokinetic mobility measurements. Since tPA has its isoelectric point (IEP) at pH 8, surface properties of SPIONs are expected to change during immobilization, as presented in Figure 4. Carboxymethylated SPIONs showed a strongly negative ζ potential and reached an IEP at around pH 2. The addition of tPA shifted the IEP of SPION^Dex−COOH^ towards pH 4. This effect was more pronounced for covalently grafted proteins than for adsorbed ones and was highest for 0.5 mg/mL covalently immobilized tPA.

### 2.3. Activity of tPA

**Figure 8:**
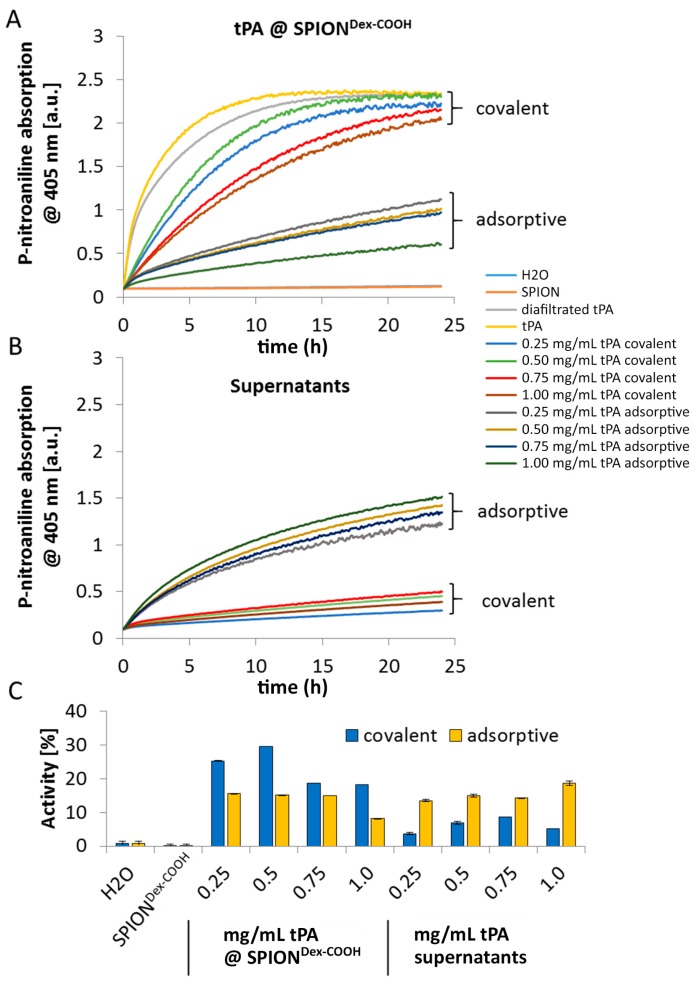
(**A**) S-2288 activity assay of covalently linked and adsorbed tPA on SPION^Dex−COOH^; (**B**) as well as the relative supernatants of the reaction mixes measured for 24 h; (**C**) Activity retention of covalently bound and adsorbed tPA on carboxymethylated SPIONs in relation to free tPA measured with the S-2288^TM^ substrate. Shown are the mean values of *n* = 3 with standard deviations.

To evaluate the remaining activity of immobilized tPA, two different assays were performed: First, the S-2288^TM^ assay, which measures the amidolytic activity towards chromogenic substrate and second, fibrinolytic activity in fibrin-containing agarose gels. Figure 8 shows the relative tPA activities determined by the S-2288^TM^ assay using equation 1 (see Methods), resulting from a cleavage of the Arg-Val bond of the assay substrate by tPA. Compared to pure and diafiltrated tPA formulation, the measurements of SPION^Dex−COOH^ with covalently grafted tPA showed a remaining activity of about 25% to 30% for initially added tPA concentrations of 0.25 and 0.5 mg/mL, respectively (Figure 8A,C). At the same tPA concentrations, the adsorptive approach had only a remaining activity of about 15%. Interestingly, the activity of the supernatant of the adsorptive approach had a similar activity of approx. 15% for all tPA concentrations (Figure 8B,C). In case of covalent immobilization, the supernatants exhibited only minimal activity below 10%.

**Figure 9:**
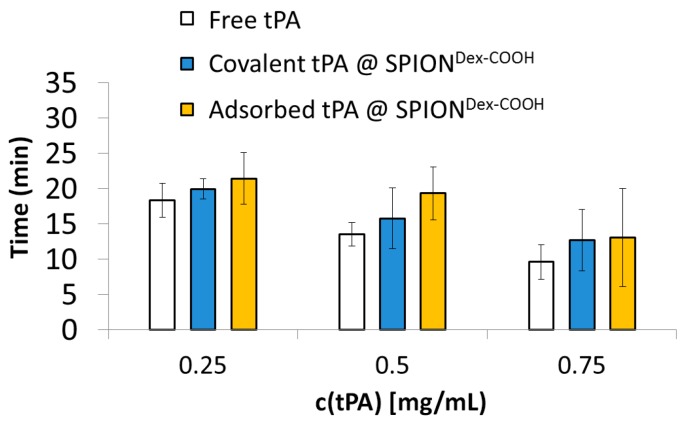
An increase in tPA concentration leads to a shorter required time for the dissolution of blood plasma clots. Water and particles without tPA served as a negative control. Since no dissolution was observed after five hours, they are not included in the figure for better visibility of the results. Shown are the mean values of *n* = 3 with standard deviations.

A different approach to investigate the tPA activity in the presence of serum was used based on the dissolution of plasma clots (Figure 9). An increase in tPA activity is characterized by a reduction in time to dissolve the clot. Thereby, with increasing tPA concentration, the required time was reduced for all samples. A comparison between free and immobilized tPA showed a reduced but still remaining activity, whereby the adsorptive approach required slightly more time for dissolution. It is noteworthy that water and particles without tPA did not cause clot dissolution within 5 h.

**Figure 10:**
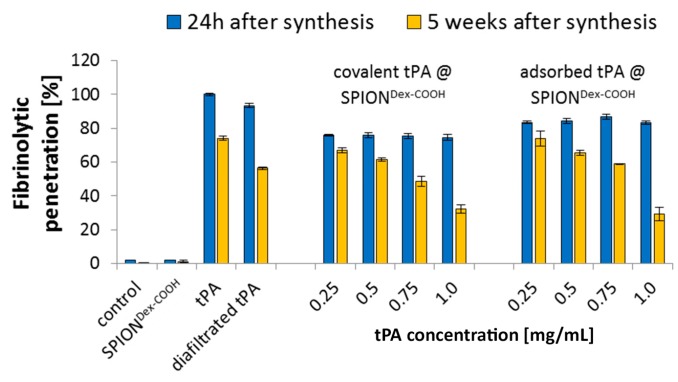
Long-term stability of covalently bound and adsorbed tPA on SPION^Dex−COOH^ determined with agarose-fibrin matrices. Shown are the mean values of *n* = 3 with standard deviations.

Interestingly, these differences in tPA activity were not detectable within agarose-fibrin matrices (Figure 10), where tPA was able to diffuse into the matrix and lyse the fibrin networks. At 24 h after synthesis, covalently bound tPA retained about 75% fibrinolytic activity, whereas adsorbed proteins showed up to 84% of the original tPA activity in thrombus-mimicking gels.

According to the manufacturer’s information, tPA is stable for 24 h after dissolution at 2–8 °C. In fact, the time span between fabrication and potential application of SPION^Dex−COOH−tPA^ is significantly longer. To examine the stability of the produced particles (covalent and adsorptive ones), the amidolytic activity (weekly [16]) as well as the fibrinolytic activity (begin and end of storage time; Figure 10) were recorded while storing the samples at 4 °C for 5 weeks. After this storage, the lysis of fibrin networks showed only a slight decrease in the tPA activity at lowest tPA concentrations, in both the covalent and adsorptive approach. Covalently bound tPA still possessed 69% of the original activity and adsorptively bound tPA had 73% activity retention. Increased protein load on the particles led to a greater loss of activity upon storage (Figure 10). Complementary measurements with the S-2288^TM^ assay confirmed the potential longer activity of the bound tPA versus free drug [16].

**Figure 11:**
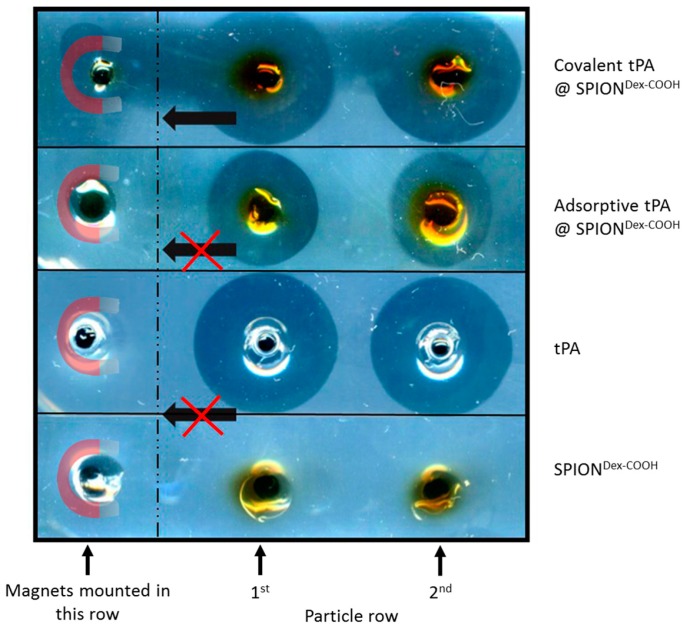
While covalently bound tPA on SPION^Dex−COOH^ effectively dissolved agarose-fibrin matrices in the direction of the external magnetic field, the adsorptive approach led only to a dissolution around the holes.

Further experiments recording the fibrinolytic activity under the influence of a magnetic field were performed for tPA-loaded SPIONs within fibrin-containing agarose plates. Cylindrical magnets with a flux density of 0.4 T were placed on the left hand side of the plate, under the slots neighboring tPA-loaded SPIONs, and the whole setup was incubated overnight at 37 °C. Figure 11 clearly demonstrates that SPIONs with covalently immobilized tPA were successfully directed towards the magnet into the fibrin matrix, leading to fibrin fiber digestion. Since the magnetic field decreases with distance to the magnet, nanoparticles filled in the holes in the 2nd row were less attracted, but still showed fibrinolysis towards the magnet. In contrast to this, the fibrinolytic areas for particles with adsorbed tPA were not directed towards the magnet, but instead formed circles around the wholes. Control samples without tPA were also attracted by the magnets but did not show any activity.

### 2.4. Cytocompatibility

**Figure 12:**
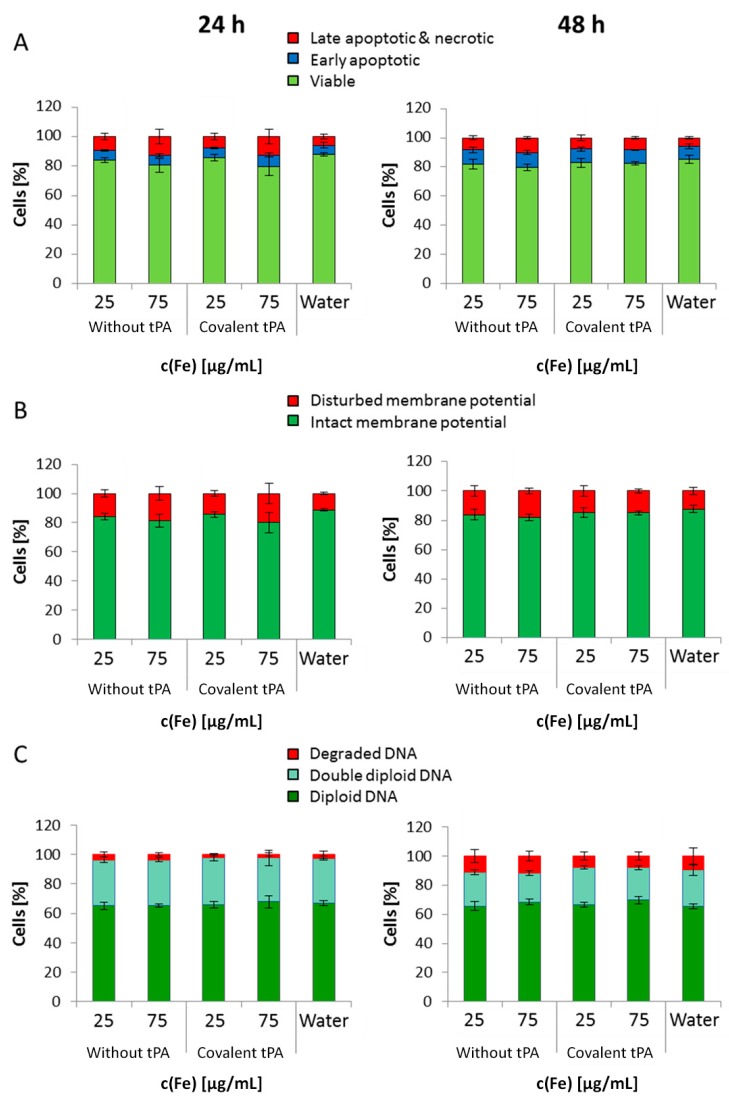
Cellular effects of tPA-free and tPA-containing SPION^Dex−COOH^. The tPA concentration corresponding to 25 µg Fe/mL is 5.75 µg/mL, and for 75 µg Fe/mL, it is 17.25 µg/mL. (**A**) Cell viability (annexin V FITC/propidium iodide (AxV/PI)); (**B**) mitochondrial plasma membrane potential integrity (1,1′,3,3,3′,3′-hexamethylindodicarbocyanine iodide (DiI)) and (**C**) DNA cycle and DNA degradation (Propidium iodide-Triton-X (PIT)) were analyzed for 24 h (left) and 48 h (right) of incubation. Shown are the mean values of *n* = 3 with standard deviations. No significant difference according to Student’s *t*-test.

In order to provide initial information concerning the cytocompatibility of the synthesized SPIONs (with and without tPA), the cellular response, the toxicity and cellular uptake were investigated in human umbilical vein endothelial cells (HUVECs). Taking the above-described results into account, SPION^Dex−COOH−tPA^ (2 mg Fe/mL with 0.5 mg/mL of covalently immobilized tPA), which showed the best fibrinolytic activity, were chosen for the experiments with HUVECs. As control particles, SPION^Dex−COOH^ were tested. Staining with annexin V FITC/propidium iodide (AxV/PI) (Figure 12A) was used as a marker for early apoptosis (AxV positive, PI negative) as well as late apoptosis and necrosis (PI positive). Following 24 h exposure, no endothelial cytotoxicity of SPIONs was detected up to the highest tested concentration, with cell viabilities (Ax negative, PI negative) of approx. 85%. The same trend was observed in the staining with 1,1′,3,3,3′,3′-hexamethylindodicarbocyanine iodide (DiI) (Figure 12B), which was employed to analyze the mitochondrial cell membrane potential. After 48 h of treatment, the potential was not altered, indicating good biocompatibility of the particles, even after tPA immobilization. Propidium iodide-Triton-X (PIT) staining (Figure 12C) was additionally performed to obtain information about the cell cycle and the DNA degradation [21]. Using this staining, a negligible increase in DNA degradation after 48 h exposure was detected in SPION-treated cells compared to the control (Figure 12C). In addition, increasing cell numbers indicated that HUVEC proliferation was not affected by the particles.

**Figure 13:**
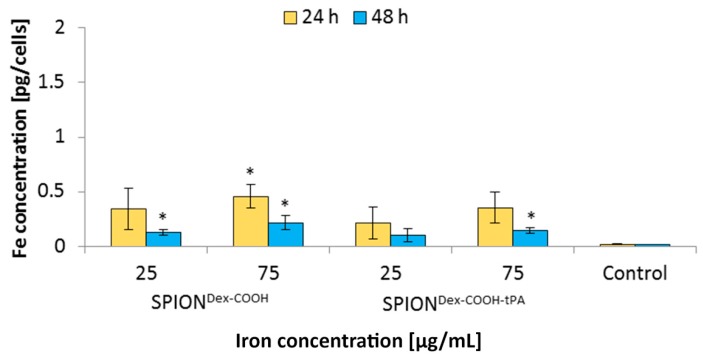
Iron uptake of SPION^Dex−COOH^ and SPION^Dex−COOH−tPA^ in HUVECs measured with microwave plasma atomic emission spectroscopy (MP-AES) after 24 and 48 h of incubation. Iron concentrations were normalized to the cell number. Shown are the mean values of *n* = 3 with standard deviations. Asterisks mark significance levels determined by Student’s *t*-test (* *p* < 0.05).

HUVECs were also used to investigate and compare the cellular particle uptake. For this purpose, HUVECs were incubated with iron concentrations of 25 and 75 µg/mL for 24 and 48 h, respectively. For both particles, a marginal cell uptake, with cellular iron concentrations lower than 0.5 pg/cell, was detected (Figure 13). After tPA binding, the particle uptake seemed to be slightly reduced, but the differences between SPION^Dex−COOH^ and SPION^Dex−COOH−tPA^ were not significant. Prolongation of the incubation time from 24 to 48 h did not lead to a higher SPION uptake; instead, the iron concentration within the cells was slightly reduced.

## 3. Discussion

Targeted delivery of tPA is the focus of current research [22]. Several magnetically controllable tPA loaded nanoparticles have been developed so far, including silica-coated magnetic nanoparticles [23], and magnetic nanoparticles coated with chitosan [24] or polyacrylic acid [25]. In a previous approach, we developed a system based on polyacrylic acid-co-maleic acid-coated SPIONs [26]. In contrast to these studies, we changed the basis to dextran-coated SPIONs because of their low internalization into cells as well as remarkable biocompatibility [27] and compared the binding efficacy and activity of covalently grafted tPA with adsorbed tPA.

Crosslinked dextran led to a very effective colloidal stabilization of the magnetite nanoparticles, which showed a nearly spherical morphology and formed a branched chain-like structure in TEM measurements due to the adsorbed dextran shell [28]. Precipitation of iron salts in the presence of the polysaccharide promoted small SPION core sizes due to confinement effects during crystal growth. The size distributions were quite narrow and in the same order of magnitude, independent of the dextran content, functionalization and tPA bonding. This may be explained by a quick and homogeneous distribution of the iron salts and the added ammonia within the dextran matrix during the cold gelation process. Furthermore, it can be assumed that the interaction of SPIONs with dextran prevented intense Ostwald ripening, as the polymer confines the spaces within which the iron oxide crystals can grow [20]. The magnetic properties of the particles are an important factor for the efficacy of the targeting approach. The synthesized particles showed no hysteresis and no remanence, indicating their superparamagnetic behavior. The measurements also demonstrate that the functionalization and the tPA binding procedure had no influence on the magnetic properties. The lack of residual magnetization reduces the probability of agglomeration, thereby avoiding uptake by phagocytes, as well as the risk of thrombosis [29].

The ζ potential of carboxymethylated SPIONs lies well above an absolute value of 30 mV at physiological pH. In addition, particles revealed a very good colloidal stability, and their hydrodynamic sizes remained in the same order of magnitude over several weeks. This indicated that stabilization was achieved by both electrostatic and steric repulsion.

Comparing hydrodynamic sizes, a 3-fold increase was observed after functionalization of SPION^Dex^ with carboxyl groups. During synthesis at a strong alkaline pH, hydroxyl groups of dextran as well as carboxyl groups are deprotonated. Hence, they repel each other, leading to a different convolution of the dextran coating and a reduction of steric stabilization efficacy. Additionally, the bonding of COOH– groups may disturb the steric stabilization by unfolding parts of the dextran shell. An increased ionic strength can significantly influence the particle’s surface, which facilitates the formation of agglomerates and may explain the increase in diameter after functionalization. tPA linking slightly increased the particle size, which may be a first hint of successful binding, but dynamic light scattering measurements are not precise enough to consider this result as proof. As tPA has a zwitterionic character and a more positive IEP, its addition shifted the IEP of SPION^Dex−COOH^ towards pH 4. This effect was more pronounced for covalently grafted proteins than for adsorbed ones and was highest for 0.5 mg/mL covalently immobilized tPA. This was in good accordance with other results and also serves as an indicator of successful immobilization of the protein on magnetite nanoparticles [30,31]. It is important to note that the stabilizing agents of commercially available tPA formulations, in this case Actilyse^®^, impede the linking to SPIONs. Actilyse^®^ is stabilized by arginine, which contains at least two free primary amine groups that can react with the o-acylisourea esters during the EDC/NHS binding reaction, lowering the amount of bound tPA [26]. To prevent this, the protein was diafiltrated prior to the binding experiments with SPION^Dex−COOH^.

As shown using gel electrophoresis, almost all added protein (up to 98%) was covalently linked to the functionalized SPIONs. Also, in the case of adsorbed protein, similar values were found. The latter outcomes are attributed to a spontaneous adhesion of active tPA onto the iron oxide surface [32]. In this case, interaction forces like hydrophobic interaction, ionic or electrostatic bonding and hydrogen bonding as well as energetic or entropic considerations may be responsible for the high binding efficiency [33,34]. The remaining activity of tPA after the different binding mechanisms was investigated via the S-2288^TM^ assay, since this activity assay reveals better accuracy for quantifiable results compared to the gel. Despite similar binding efficiencies obtained from electrophoretic gel analysis, the difference in remaining activities indicates binding of different amounts of tPA to SPIONs after the covalent and adsorptive approach. In the covalent approach, higher activity was found on the particles compared to the supernatant. In contrast to this, the adsorptive approach led to similar activities on the particles and in the supernatant. This suggests that in this case, tPA not strongly adsorbed on the particle surface can easily desorb. For both approaches, lower values in relation to free tPA may be explained by partial linking to active sites of the protein or interconnection of protein molecules among each other. In addition, the activity of diafiltrated tPA decreased slightly relative to free tPA, due to a preferential adsorption of the protein to the surface of the filter membrane and hose systems [26]. The reduction of tPA activity with increased protein quantity may suggest that binding efficiencies were higher for lower amounts of initially added tPA. This may be attributed to a reduced hindrance, crosslinking, or interactions between the protein molecules. After binding tPA to the coated particles, Ma et al. [25] reported also a decline of fibrinolytic tPA activity in comparison to free tPA, which is in good concordance with our dextran-coated SPIONs containing tPA. Chen et al. [23,24] immobilized tPA on the particle surfaces with almost full activity retention for low tPA amounts, which also decreased at higher tPA loadings. In addition, tPA activity was also assessed by dissolution of plasma clots. The experiments show that the tPA activity is not hampered by the presence of serum.

In contrast to the S-2288^TM^ assay, almost no differences in tPA activities between the examined particles were observable in agarose-fibrin matrices. Higher fibrinolytic activity values compared to amidolytic activities may be caused by a longer incubation time compared to the S-2288^TM^ assay. Hence, tPA has more time for reaction with the fibrin network, which may be more easily dissolved by the protein. In the fibrinolytic assay, tPA activity values were even higher for adsorbed protein. This finding may be explained by a partial linking to active sites of tPA or to conformational changes of the protein in the case of the covalent approach. Furthermore, adsorbed tPA is more easily released from the SPIONs, which may also be beneficial for thrombolysis, if particles are administered close to the thrombus. It is assumed that adsorptively bound tPA is most likely released from the particle surface and easily diffuses into the agarose-fibrin matrix. Nevertheless, SPIONs with covalently bound tPA seem to be more efficient in magnetic drug targeting, as the magnetic nanoparticles can be guided to and into a fibrin-matrix by a magnet without loss of the protein.

In general, SPION^Dex−COOH^ and SPION^Dex−COOH−tPA^ showed almost imperceptible cellular uptake over 24 and 48 h with the resulting cellular iron concentrations lower than 0.5 pg/cell. Upon tPA binding, the uptake seemed to be slightly but not significantly reduced. Other SPIONs, e.g., which are coated with lauric acid and bovine serum albumin, have a 20-times higher uptake in HUVECs under the same conditions [35]. Based on these results, it can be assumed that the tPA bonding does not influence the cellular uptake of SPIONs. This low internalization of particles by cells was probably also responsible for the observed lack of their cytotoxicity. Flow cytometry analyses, investigating the mitochondrial membrane potential, the increase of DNA degradation and the cell cycle alteration, did not indicate any major changes in the presence of the SPIONs. Even at the concentration of 75 µg Fe/mL, their impact on HUVEC viability was very low, indicating good cytocompatibility under the examined conditions.

For application in the human body, the covalent approach seems to be more advantageous as the SPIONs can be moved in the direction of a magnetic field while simultaneously dissolving a fibrin network. Chen et al. [23,24] and Ma et al. [25] previously performed tissue perfusion and thrombus dissolution experiments in in vivo models. Our present in vitro findings are in good accordance with the results of these studies, showing that tPA grafted on the coated magnetite nanoparticles may be an effective tool for thrombus dissolution in a shorter time, or at lower doses.

Based on our present data, dextran-coated SPIONs containing 0.5 mg/mL tPA may, even after weeks of storage, represent a suitable candidate for targeted thrombolytic therapies under an external magnetic field.

## 4. Materials and Methods

### 4.1. Materials

In all protein crosslinking experiments, rtPA (Actilyse^®^) from Boehringer Ingelheim (Boehringer Ingelheim, Ingelheim am Rhein, Germany) was used. Iron(II)chloride tetrahydrate (FeCl_2_·4H_2_O), iron(III)chloride hexahydrate (FeCl_3_·6H_2_O), epichlorohydrin (ECH), N-hydroxysuccinimide (NHS), 2-(*N*-morpholino)ethanesulfonic acid hydrate (MES), 4-(2-hydroxyethyl)piperazine-1-ethanesulfonic acid (HEPES), phosphate buffered saline (PBS), propidium iodide (PI), fibrinogen human type I from human plasma, ribonuclease A from bovine pancreas and thrombin from human plasma were purchased from Sigma Aldrich (Taufkirchen, Germany). T-40 Dextran, hydrochloric acid, 25% ammonia solution (NH_3_), silver nitrate, 1-ethyl-3-(3-dimethylaminopropyl)carbodiimide (EDC), 30% acrylamide/bisacrylamide solution, agarose low melt, ammonium persulfate (APS) bromophenol blue, sodium dodecyl sulfate (SDS), sodium hydroxide (NaOH), Triton X-100, sterile and unsterile Rotilabo^®^-syringe filters with cellulose mixed ester (CME) membrane (diameter: 25 mm, pore size 0.22 µm) and Tris-hydrochloride (Tris-HCl) were obtained from Carl Roth (Karlsruhe, Germany). Chromogenic substrate S-2288 TM was purchased from Chromogenix (West Chester, OH, USA), Ringer solution from Fresenius Kabi AG (Bad Homburg vor der Höhe, Germany) and Tris-buffered saline (TBS) from BioRad (Hercules, CA, USA). Annexin V-FITC, Hexamethylindodicarbo-cyanine iodide dye (DilCI(5)), and Hoechst 33342 (Hoe) were obtained from Thermo Fisher Scientific (Waltham, MA, USA). MUSE^®^ cell & viability dye was purchased from Merck KGaA (Darmstadt, Germany) and TEM grids (Athene S 147-2) from Plano (Wetzlar, Germany). Extracellular growth medium (ECGM) with the corresponding SupplementMix and HUVECs were obtained from PromoCell (Heidelberg, Germany). Vivaspin 20 and 500 ultrafiltration units (100 kDa) were obtained from Sartorius AG (Göttingen, Germany). Modified polyethersulfone membrane (30 kDa) and SPECTRA/POR^®^ 6 dialysis tubing (8–10 kDa) were obtained from Spectrum Laboratories Inc. (Milpitas, CA, USA). For all experiments, water was deionized and filtered by an ultra-pure purification unit (Siemens Ultra Clear, Germany).

### 4.2. Preparation of Carboxymethylated Dextran-Coated Spions

Dextran-coated SPIONs (SPION^Dex^) were synthesized in a cold gelation process as previously described [15]. Briefly, an aqueous dextran solution was mixed with an aqueous solution of FeCl_3_∙6H_2_O and FeCl_2_∙4H_2_O (molar ratio of 2:1) and filtered through a 0.8 µm membrane into a three-neck flask. The yellow dispersion was stirred at 4 °C under argon atmosphere. Prior to heating at 75 °C, 25% NH_4_OH was added dropwise to the solution under rapid stirring, which resulted in precipitation of SPIONs, indicated by the black color of the solution. In order to remove excess ions and excess dextran, the particles were purified by dialysis against water and ultrafiltration. The dextran shell of the particles was then crosslinked using epichlorohydrin. After purification, SPION^Dex^ were further functionalized with carboxylic acid groups [36]. In short, 10 M NaOH was added to the nanoparticle suspension to adjust the pH to 12.4. After cooling in an ice-bath, monochloroacetic acid was added to the particles, and the dispersion was stirred at 60 °C for 90 min. Neutralization with acetic acid, purification by dialysis against water and ultrafiltration resulted in SPION^Dex−COOH^. After purification of the particles, they were sterile filtered and stored at 4 °C until further use.

### 4.3. tPA Binding

Commercially available tPA is stabilized by l-arginine to enhance its solubility and to prevent aggregation. Prior to the reaction, it was necessary to remove this arginine excess, as it disturbs the bonding of tPA to SPION^Dex−COOH^. For this purpose, a tangential flow filtration unit (SpectrumLabs, Rancho Dominguez, CA, USA) with modified polyethersulfone hollow fiber filters, with a molecular weight cut-off of 30 kD, were used [26]. In this step, the lyophilized protein was dissolved in 0.3 M HEPES at pH 7, which was also used as filtration buffer. At a feed flow of 15 mL/min, at least 30 g permeate was obtained. The tPA solution was set to its original volume and stored at 4 °C.

For the subsequent carbodiimide coupling of tPA to carboxymethylated nanoparticles, a slight excess of EDC/NHS with a molar ratio of 1:2 was used. EDC (7.76 mg/mL) was prepared in 0.1 M 2-(*N*-morpholino)ethanesulfonic acid (MES) buffer at pH 6.3 and mixed with NHS in the dark. Then, the EDC/NHS mixture was added to SPION^Dex−COOH^ (2 mg Fe/mL) at a volume ratio of 2:1 in 0.1 M MES and stirred in the dark at 4 °C. Afterwards, the unreacted EDC/NHS was removed by ultrafiltration in Vivaspin 500 concentrators. These washing steps were repeated three times, whereby 200 µL MES buffer was added after each step. The activated nanoparticles were stirred for a further three hours with the addition of 250 µL purified tPA. For separating the nanoparticles from the supernatant of the reaction, the dispersion was centrifuged, and the tPA-loaded particles (SPION^Dex−COOH−tPA^) were redispersed in distilled water and stored at 4 °C. The supernatants were later used for gel electrophoresis to determine the immobilized tPA amount. For comparison, experiments were also done in the absence of EDC/NHS. Instead, 500 µL 0.1 M MES was used, whereby tPA was only adsorbed and not covalently bound to the nanoparticles.

### 4.4. Particle Size and ζ Potential Measurement

The mean hydrodynamic diameter of the nanoparticles was determined via DLS using a Nanophox (Sympatec, Clausthal-Zellerfeld, Germany) cross-correlation spectrophotometer. pH-dependent ζ potential measurements were performed in aqueous solution with a Stabino^®^ (Particle Metrix, Inning, Germany) in the range of pH 2 to 11 by the addition of 0.02 M HCl and 0.02 M NaOH, respectively. The experiments were performed in triplicates, and the results were averaged.

### 4.5. Transmission Electron Microscopy

TEM images of the magnetic nanoparticles were taken with a Philips CM300 UltraTWIN transmission electron microscope (Philips, Eindhoven, The Netherlands), which was operated at 300 kV in imaging mode. The volumes of 20 µL of the highly diluted nanoparticle dispersions were dropped on a carbon-coated copper TEM grid (Athene^®^ S 147-2, Plano, Wetzlar, Germany) and the TEM images were analyzed using ImageJ software to determine the respective particle sizes (*n* = 100) and distributions.

### 4.6. Determination of the Iron Content

The total iron content of the nanoparticles was determined with an Agilent 4200 microwave plasma-atomic emission spectrometer (Agilent Technologies, Santa Clara, CA, USA). For this purpose, particles were dissolved with HNO_3_ at 95 °C for 10 min. After cooling to room temperature, samples were diluted with water. Then, the iron content was measured using a commercial iron standard solution for calibration with iron concentrations ranging from 0.05 to 5.0 mg/L. The iron measurements in the aliquots were performed in triplicate, and the results were averaged.

### 4.7. Magnetization Measurements

Magnetic properties of the nanoparticles were measured using a superconducting quantum interference device (SQUID)-based susceptometer (QD-MPMS-XL-5, Quantum Design Inc, San Diego, CA, USA). The magnetization of the MNP dispersions (75 µL within a polycarbonate capsule) was measured within applied fields ranging from 0 to 4000 kA/m. Further details can be found in Eberbeck et al. [37].

### 4.8. Fourier Transform Infrared Spectroscopy

The chemical structure of the lyophilized specimen was detected with a Bruker ALPHA FTIR spectrometer (Bruker Corporation, Billerica, MA, USA) in attenuated total reflection mode with an excitation wavenumber in the mid-infrared region from 400 to 4000 cm^−1^ and a step size of 0.5 cm^−1^.

### 4.9. Investigation of tPA Loading Efficiency

The supernatants of the particles were collected, and the amount of unbound tPA was determined in silver stained electrophoretic gels. Briefly, 25 µL of the supernatants and the tPA standard were denatured in 6× SDS-PAGE sample loading dye (G-Biosciences, St. Louis, MO, USA). Heating at 95 °C for 5 min was followed by electrophoretic separation within a 10% SDS polyacrylamide gel at 30 mA. For silver staining, gels were incubated in a fixation solution (60% ethanol, 0.05% acetic acid), washed thrice in 50% ethanol and sensitized within 0.02% sodium thiosulfate pentahydrate. The following was conducted until staining was sufficient: washing with distilled water, incubation in silver solution (10 mM AgNO_3_, 0.075% formaldehyde) for 20 min in the dark, washing with water and developing (0.6 M Na_2_CO_3_, 0.05% formaldehyde in water). Washing twice with distilled water and storing in the stopping solution (0.4% distilled water, 0.4% ethanol, 0.2% acetic acid) finished the procedure. The stained gels were photographed, and protein bands were quantified using ImageJ software. The experiments were performed in triplicate, and the results were averaged.

### 4.10. Determination of tPA Activity with S-2288™

The amidolytic activity of tPA was measured with the chromogenic substrate S-2288^TM^ (Chromogenix, no. 82085239). All specimens, respective supernatants and freshly prepared tPA stock solution (1 mg/mL in water) were diluted to 1:50 with water. Each solution (50 µL) was pipetted in triplicate into a 96-well cell culture plate and mixed with 1 mM assay buffer (S-2288^TM^ in 0.1 mM Tris-HCl pH 8.4). The absorption was recorded at 405 nm in a spectrophotometer (FilterMax F5, Molecular Devices, Sunnyvale, CA, USA) for 24 h (interval: 5 min), and the kinetic activity was determined as the slope of the regime where the correlation between the change in absorbance (ΔA) and test time *t* in minutes was linear:

(1) $$\mathrm{tPA}\;\mathrm{activity}=\frac{\Delta A}{t}$$

The experiments were performed in triplicate, and the results were averaged.

### 4.11. Dissolution of Plasma Coagulates

Platelet-poor human plasma was obtained from freshly drawn whole blood of human volunteers that was anticoagulated with sodium citrate by centrifugation for 10 min at 2500 *g*. Aliquots of the plasma were pipetted into preheated cuvettes containing a metal ball. Then, thrombin was added to initiate plasma coagulation. Once the plasma was completely solid, free tPA or particle-bound tPA was added, and the time until the thrombus was dissolved was determined with a Merlin MC4 coagulometer (ABW Medizin und Technik GmbH, Lemgo, Germany). Water and particles without tPA served as the negative control. The experiments were performed in triplicate, and the results were averaged.

### 4.12. Determination of tPA Activity with Fibrin-Agarose Gels

Fibrin-containing agarose gels were prepared to analyze the fibrinolytic tPA activity by fibrin clot lysis. For this purpose, plates were assembled by mixing 1% agarose solution in tris-buffered saline (TBS) with 5 U thrombin (dissolved in 1 mL distilled water) and 12.5 mL fibrinogen solution (3 mg/mL in TBS) at 37 °C. The mixture was then poured into a well plate cover. A stamp with 24 cylindrical holes was placed into the fibrin-agarose solution before incubation at 37 °C for 3 h and subsequent cooling at 4 °C. After removing the stamp, the created holes were filled with 20 µL sample and incubated at 37 °C overnight. Comparison of the fibrinolytic zones around the holes allowed an estimation of the degree of clot lysis by still active tPA. Measurements were performed in triplicate, and the results were averaged.

### 4.13. Storage Stability of Free and Immobilized tPA

In order to analyze how the activities of SPIONs, SPIONs loaded with tPA and free tPA change with time, all samples were stored at 4 °C for 5 weeks after production, and S-2288^TM^ assays as well as fibrin-agarose gels were repeated during this time. The amidolytic tPA activity was recorded every week and compared to the activity at 24 h post-synthesis. tPA activity in fibrin-agarose gels was examined at 24 h and after 5 weeks of storage. The experiments were performed in triplicate, and the results were averaged.

### 4.14. Cell Cultivation

Primary human umbilical vein endothelial cells were purchased from PromoCell (Heidelberg, Germany). To investigate the in vitro toxicity of the magnetic nanoparticles, HUVECs of passage 3 were cultivated in enhanced endothelial cell growth medium at 37 °C, 95% humidified air and 5.0% CO_2_. For further passaging, trypsinization was performed according to Friedrich et al. [38].

### 4.15. Cell-Based Experiments

HUVECs were counted and adjusted to a concentration of 2.4 × 10^4^ cells per ml in cell culture medium. Then, 2.5 mL of the cell suspension was seeded into each well of a 6-well plate. After 24 h, sterile filtered SPIONs with and without tPA were added to the cells to final iron concentrations of 25 or 75 µg/mL in cell culture medium. Treated cells were incubated for 24 and 48 h, followed by harvesting, washing twice with PBS and resuspending in 0.5 mL PBS. HUVEC suspensions were used for cytotoxicity analysis by flow cytometry as well as SPION quantification measurements using MP-AES and for the determination of the absolute cell number and viability with Muse^®^ Cell Analyzer (Merck-Millipore, Billerica, MA, USA). The experiments were performed in triplicate, and the results were averaged. Significance was determined with Student´s *t*-test.

### 4.16. Flow Cytometry and Particle Uptake

Flow cytometry was performed using a Gallios cytofluorometer (Beckman Coulter, Brea, CA, USA) in order to investigate cell morphology and cell viability. Therefore, 50 µL aliquots of each cell suspension and 250 µL freshly prepared 4-color staining solution, containing 2 µL/mL PI, 1 µL/mL AxV-FITC, 1 µL/mL Hoe and 0.4 µL/mL DiI in Ringer’s solution, were mixed and incubated for 20 min at 4 °C. For cell cycle analysis and DNA degradation, another 230 µL cell suspension was fixed with 3 mL 70% (*v*/*v*) ice-cold ethanol and stored at −20 °C. Centrifugation for 5 min at 400 rcf (relative centrifugal force), removing of the supernatant, washing with PBS and resuspending in 0.5 mL PBS and 0.5 mL DNA extraction buffer (192 mL of 0.2 M Na_2_HPO_4_ in 8 mL 0.1% Triton X-100 (*v*/*v*)) for 5 min at room temperature succeeded. Cells were centrifuged again, and the supernatant was removed before 400 µL PIT DNA staining solution (20 µg/mL PI and 0.2 mg/mL DNase free RNase (cat. no.: R6513; in PBS) was added, and incubation for 30 min in the dark followed. All flow cytometric experiments were conducted in triplicate, data assessment was performed with Kaluza software version 1.2 (Beckman Coulter) and the results were averaged. In addition, the absolute iron content of all samples was determined using the above-described MP-AES. Cell pellets from a 200 µL cell suspension were dissolved in 65% HNO_3_ at 95 °C and after cooling, the volume was adjusted to 0.5 mL with distilled water. The iron content was normalized to the total HUVEC cell number of each pellet, measured by Muse^®^ cell counting. The experiments were performed in triplicate, and the results were averaged. Significance was determined with Student´s *t*-test.

## 5. Conclusions

To produce a magnetically guided drug delivery system for thrombolytic therapy, dextran-coated SPIONs were successfully synthesized and functionalized with carboxyl groups, followed by grafting with tPA. FTIR measurements, as well as the zeta potential analysis, qualitatively confirmed the chemical alteration of surface structures upon tPA bonding. Up to 98% of the initially added tPA amount was efficiently bound to SPION^Dex−COOH^. Both covalently linked and adsorbed tPA on carboxymethylated SPIONs displayed a high performance in thrombus-mimicking fibrin-containing agarose gels, yielding fibrinolytic activity values of 75% and 84%, respectively. Even after 5 weeks of storage, both particle types retained most of their original activity dependent on the initial tPA concentration. SPION^Dex−COOH^ with covalently linked tPA were magnetically attractable in fibrin-agarose gels, while the protein locally dissolved the fibrin network in the direction of the magnetic field. Because of good biocompatibility with primary human endothelial cells, these particles constitute a promising candidate for further magnetic drug targeting studies and a potential future use for a more effective local thrombolysis.

## Figures and Tables

**Table 1:** Hydrodynamic size, ζ potential and pH values of functionalized and tPA-loaded (tPA concentration: 0.5 mg/mL) SPIONs.

Condition	Sample	Hydrodynamic Diameter in nm	ζ Potential in mV	pH
Water	SPION^Dex^	65 ± 3	−1 ± 0.3	7.8
	SPION^Dex−COOH^	173 ± 9	−58 ± 4	7.0
MES buffer	SPION^Dex−COOH^	166 ± 4	−48 ± 2	6.7
	SPION^Dex−COOH−tPA^	170 ± 6	−46 ± 2	6.5

Shown are the mean values of *n* = 3 with standard deviations. SPION^Dex^: dextran-coated SPIONs MES: 2-(*N*-morpholino)ethanesulfonic acid.

## Abbreviations

AxV	Annexin V
DiI	Hexamethylindodicarbocyanine iodide dye
DLS	Dynamic light scattering
EDC	1-Ethyl-3-(3-dimethylaminopropyl)carbodiimide
Fe_3_O_4_	Magnetite
FITC	Fluorescein isothiocyanate
FTIR	Fourier transform infrared spectroscopy
HEPES	4-(2-hydroxyethyl)piperazine-1-ethanesulfonic acid
Hoe	Hoechst33342
HUVEC	Human umbilical vein endothelial cells
IEP	Isoelectric point
MES	2-(*N*-morpholino)ethanesulfonic acid
MP-AES	Microwave plasma atomic emission spectroscopy
NHS	*N*-Hydroxysuccinimide
PBS	Phosphate buffered saline
PI	Propidium iodide
SDS-PAGE	Sodium dodecyl sulfate polyacrylamide gel electrophoresis
SPION	Superparamagnetic iron oxide nanoparticle
SPION^Dex^	Dextran coated SPION
SPION^Dex−COOH^	Carboxymethylated SPION^Dex^
SPION^Dex−COOH−tPA^	SPION^Dex−COOH^ with covalently grafted tPA
SQUID	Superconducting quantum interference device
TEM	Transmission electron microscopy
